# Traumatic Vertebral Artery Injury: Diagnosis, Natural History, and Key Considerations for Management

**DOI:** 10.3390/jcm14093159

**Published:** 2025-05-02

**Authors:** Ben Teasdale, Edwin Owolo, Varun Padmanaban, Aladine A. Elsamadicy, Abdelaziz Amllay, Ganesh M. Shankar, Penina P. Krieger, Robert W. Regenhardt, Ryan M. Hebert, Christopher J. Stapleton, James D. Rabinov, Charles C. Matouk, Aman B. Patel, Nanthiya Sujijantarat

**Affiliations:** 1 Department of Neurology, Mass General Brigham, Harvard Medical School, Boston, MA 02114, USA; bteasdale@mgb.org (B.T.); pkrieger@mgb.org (P.P.K.); 2 Department of Neurosurgery, Mass General Brigham, Harvard Medical School, Boston, MA 02114, USA; eowolo@mgh.harvard.edu (E.O.); vpadmanaban@mgh.harvard.edu (V.P.); cstapleton@mgh.harvard.edu (C.J.S.); jrabinov@mgh.harvard.edu (J.D.R.); abpatel@mgh.harvard.edu (A.B.P.); 3 Department of Neurosurgery, Yale-New Haven Hospital, Yale University, New Haven, CT 06510, USA; aladine.elsamadicy@yale.edu (A.A.E.); abdelaziz.amllay@yale.edu (A.A.); gshankar@mgh.harvard.edu (G.M.S.); ryan.hebert@yale.edu (R.M.H.); charles.matouk@yale.edu (C.C.M.); 4 Department of Neurology, University of Texas Health Science Center, Houston, TX 77030, USA; robert.regenhardt@uth.tmc.edu; 5 Department of Radiology, Mass General Brigham, Harvard Medical School, Boston, MA 02114, USA; 6 Department of Radiology, Yale-New Haven Hospital, Yale University, New Haven, CT 06510, USA

**Keywords:** blunt cerebrovascular injury, vertebral artery injury, cervical trauma, traumatic vertebral occlusion, spinal injury

## Abstract

Vertebral artery injury (VAI) is a known complication of blunt cervical spine trauma with a potential risk of stroke. Factors including cervical bony injury, spinal cord injury, and overall trauma severity have been linked to an increased risk of VAI. Despite its prevalence, there is little consensus on various aspects of this pathology, including its initial screening, diagnostic approaches, and therapeutic strategies. A recent systematic review and meta-analysis from our group highlighted the dynamic nature of vertebral artery occlusion, revealing the underrecognized recanalization rates and potential stroke risks associated with delayed recanalization. While anticoagulant and/or antiplatelet therapy (ACAP) remains the cornerstone of VAI management, treatment is often complicated by co-existing injuries, such as intracranial hemorrhage or cervical trauma, which may preclude or delay ACAP usage or necessitate surgical intervention. This comprehensive narrative review synthesizes the latest evidence on VAI and associated ischemic sequelae, with the goal of elucidating its pathophysiology and natural history, summarizing current data on screening and diagnosis, and exploring key considerations for medical and endovascular management.

## 1. Introduction

Vertebral artery injury (VAI) is a complication of blunt cervical spine trauma and is commonly classified using the Denver grading system. This system categorizes VAI into five grades: minimal dissection (grade I), intramural hematoma/dissection with 25% or more luminal narrowing (grade II), pseudoaneurysm (grade III), occlusion (grade IV), and transection (grade V) (Table 1) [1]. Broadly, arterial injuries are typically classified based on their etiology (spontaneous vs. traumatic) and mechanism (blunt vs. penetrating). Although many cases of traumatic VAI do not present with neurological deficits, ischemic stroke remains a recognized complication. This risk is believed to result from hemodynamic failure or artery-to-artery thromboembolism originating from the site of injury [2,3].

Despite its prevalence, there is little consensus on various aspects of VAI, including initial screening, diagnostic approaches, and therapeutic strategies. Because many VAI patients can present with non-specific symptoms such as headache, dizziness, or neck pain, detection of VAI can be delayed or missed altogether. In polytrauma cases, neurological assessment may be further complicated by distracting injuries that affect mental status or obscure lateralizing deficits.

While dedicated angiographic imaging is widely accepted as necessary for diagnosing VAI, debate persists regarding the appropriate clinical indications, the optimal timing of screening, and the preferred imaging modality. Once VAI is identified, management primarily aims to prevent injury progression and to mitigate ischemic complications. Treatment decisions rely on a thorough evaluation of both acute and longitudinal risks, which are influenced by various clinical and radiographic factors.

The mainstay of medical therapy for VAI involves anticoagulant and/or antiplatelet therapy (ACAP). However, in polytrauma patients with coexisting injuries such as intracranial hemorrhage or conditions requiring surgical intervention, the associated hemorrhagic risks must be carefully balanced against the potential benefits. Additionally, growing evidence suggests that VAI and its ischemic sequelae are dynamic, evolving not only as the lesion heals or progresses, but also in response to local interventions like cervical reductive surgery [4]. Assessing these risks at both the initial presentation and over time is crucial for guiding management plans, including the initiation of medical therapies and invasive procedures.

This comprehensive review aims to provide an overview of VAI and its associated complications, and to synthesize the current evidence on its pathogenesis, diagnosis, and management.

## 2. Epidemiology

The incidence of VAI among blunt trauma patients varies significantly among the studied population. In the general blunt trauma population, VAI is relatively uncommon. In a recent systematic review and meta-analysis by Michalopoulos et al., the pooled rate of VAI was reported to be 0.5% among all blunt trauma patients [5]. However, among patients specifically selected for screening with dedicated angiographic imaging such as computed tomography angiography (CTA), magnetic resonance angiography (MRA), or diagnostic subtraction angiography (DSA), the incidence was significantly higher at 15%, although the criteria for patient selection were variable between studies and were subject to clinical judgment. Notably, the I^2^ value for this pooled rate was as high as 96%, signifying substantial heterogeneity between the included studies.

For patients with identified and graded VAI, the early study by Biffl et al. reported grade I injury as the most common, accounting for 53% of cases [6]. However, when more recent studies were considered, complete vertebral artery occlusion (VAO or grade IV injury) was the most commonly identified pathology (pooled occurrence of 42%), followed by grade II (28.2%), and grade I (23.4%) [5]. This difference is likely attributed to the shift in imaging modalities, with non-invasive CTA increasingly replacing DSA in contemporary studies. Although the adoption of CTA-based screening has led to an increased detection rate in general, in the confirmed VAI cases, DSA may be more sensitive in distinguishing subtle changes in the luminal irregularities seen in lower-grade injuries.

Grade V injuries, characterized by complete vertebral artery transection, remain exceedingly rare in the context of blunt trauma. Notably, no such cases were reported in the Biffl et al. series [6]. Michalopoulos et al. identified only 6 cases among the 1277 patients included in their systematic review and meta-analysis [5].

Since VAI often reflects the severity of trauma, concurrent vascular injuries are not uncommon. Bilateral VAIs have been reported in up to 40% of patients in one series [7]. Unlike isolated VAI, bilateral VAIs are more likely to cause ischemic symptoms by way of acute hemodynamic failure, often leading to the diagnosis [3]. Additionally, carotid artery injuries are frequently found with VAI. Biffl et al. reported co-occurrence in as high as 32% of the cases, further highlighting the need for comprehensive vascular assessment in polytrauma patients [6].

Motor vehicle accidents and falls are the most commonly listed mechanism of injury leading to VAI, with Temperley et al. reporting incidences of 58% and 25% for these mechanisms, respectively [8]. However, a diverse array of mechanisms have been described, including sporting accidents, chiropractic cervical neck manipulation, and even high pillow angle [9]. In a retrospective study comparing blunt and penetrating cervical trauma, VAI was found to be more common in penetrating injuries. Blunt trauma, on the other hand, was more frequently associated with low-grade VAI (grades I and II) [10].

## 3. Associated Cervical Spine Trauma

Given the vertebral artery’s passage through the bony cervical spine, the association between cervical spine trauma and VAI is well established in the literature [11]. Among the patterns of injury associated with VAI, fractures extending into the transverse foramen were the most commonly reported as the primary cause of injury, followed by facet dislocation or subluxation [12,13,14,15,16,17]. Anatomically, injury to the V2 and V3 segments is commonly associated with bony fractures given its relationship with C2 to C6. Moreover, transverse foraminal fractures with intra-foraminal bony fragments have been found to be highly predictive of VAI [18,19].

Beyond fractures, other injury patterns frequently associated with VAI include ligamentous disruption, facet joint injury, dislocation, or subluxation. Mechanistically, VAI secondary to subluxation or flexion-extension injuries is thought to occur due to the direct compression of the artery by bony or ligamentous structures, or due to the stretching of the artery across adjacent vertebrae [20,21]. Nagata et al. further suggested that direct injury to the vertebral artery, rather than post-traumatic elongation, is more predictive of VAI, although transient shear forces present at the time of injury could not be measured [22].

The incidence of VAI appears to vary depending on the level of cervical spine involvement. In a review of 12 studies where the specific injury level was noted, Temperly et al. reported that C5 was the most frequently associated vertebra (25%), followed by C2 and C6 (20% each), C1 (12%), C4 (11%), C3 (7%), and C7 (6%) [8]. Additionally, multilevel fractures were associated with a higher risk of VAI in several studies [13,19,23].

Among the limited studies that have identified the specific segment of VAI, the V3 segment was the most commonly involved (44%) [13]. Concomitant cervical spine fractures in patients with vertebral artery dissections have been found to portend a worse prognosis [24]. The data on cervical spine injury in the context of VAI remain highly heterogeneous, and clinical outcomes may be influenced not only by the VAI itself, but also by concurrent cervical fractures, spinal cord injury, intracranial trauma, or a combination of these factors.

## 4. VAI Screening

The clinical presentation of VAI is highly variable and can present as an isolated finding or in association with a wide range of pathologies. In 1999, Biffl et al. introduced the Denver Criteria with the purpose of outlining the parameters needed to obtain an arteriographic evaluation in blunt cerebrovascular injury (BCVI) [25]. These criteria included patients with severe facial fractures (Le Fort II or III), severe traumatic brain injury (TBI) with Glascow Coma Scale (GCS) < 6, cervical spine fracture, subluxation, and/or ligamentous injury at any level, as well as near hanging with anoxic brain injury. In the same year, Biffl et al. also developed a grading system for arterial injury, classifying it into five grades (I through V), as previously outlined [1]. These criteria and grading scales were widely adopted and held implications for diagnosis, treatment strategies, and prognosis. While the original Denver criteria applied broadly to BCVI, a subsequent publication by the same group specifically detailed the incidence, mechanism, and outcomes of VAI [6]. Since then, the grading scale has been extensively used and validated in many studies specific to VAI.

The first iteration of the Denver criteria failed to detect a substantial portion of BCVI, although attempts to liberalize screening were limited by their dependence on invasive catheter-based angiography [26]. However, technical advances in the performance of CT in the early 2000s facilitated a shift from DSA to non-invasive, CTA-based diagnosis [7,25,27,28,29,30,31,32,33,34]. Eastman et al. found that this change sped up the time to diagnosis 12-fold [31]. Additionally, the broader adoption of CTA helped to streamline the initiation of medical therapy [35].

The relative ease of implementing CTA screening programs enabled the expansion of screening criteria, thereby improving sensitivity. Notably, these revised criteria refined high-risk cervical fractures, facial and cranial trauma, traumatic brain injury, and extra-cervical vascular injury [26,28]. In a large-scale validation study using a database of 258,935 trauma patients, Shibahashi et al. confirmed that a high-energy mechanism, cervical spine fracture, skull base fracture, and a low GCS score were predictors of BCVI; these were findings largely consistent with prior publications [36,37,38]. While the targeted risk factors identified at presentation have traditionally guided the need for angiographic evaluation, liberalized screening protocols have identified a significant number of patients with blunt VAI who do not present with classical risk factors [39,40,41,42,43,44,45].

A systematic review and meta-analysis examining the utilization of imaging modalities in the diagnosis of VAI found that of 3215 patients in 33 studies, CTA was the most commonly used modality (92%). In contrast, MRA was used in only 8% and DSA in just 3% [46]. In a separate systematic review and meta-analysis, Karagiorgas et al. identified five studies comparing the diagnostic performance of MRA against DSA [47]. The authors found that while MRA demonstrated a high specificity (91%), with contrasted MRA outperforming time-of-flight (TOF) technique, the sensitivity of MRA remained relatively low at 55% for VAI detection. Given the lack of clear benefit to either sensitivity or specificity, as well as the increased time required for image acquisition, MRA plays a limited role in current VAI screening practices, with most of its reported use occurring prior to the widespread adoption of CTA as the primary diagnostic modality [48,49,50,51,52]. Nonetheless, MRI remains valuable for detecting ischemic changes on follow-up imaging, or with the concurrent detection of cervical soft-tissue injuries, including ligamentous or spinal cord injury [49,53]. Reports of the use of other imaging modalities in VAI screening, such as ultrasonography, are rare and of unclear utility [53,54].

Several retrospective studies have suggested that CTA may overestimate the burden of disease in both VAI and BCVI more broadly [55,56]. Yi et al. found that a substantial number of patients initially diagnosed with VAI on CTA were later found to have normal findings on confirmatory DSA [56]. False-positive rates were particularly high in grade I lesions. The authors further reported that the number of DSAs needed to de-escalate therapy for one patient was as low as 3.2, underscoring the potential clinical impact of overdiagnosis [56].

Despite these limitations, the rapid expansion of CTA-based screening for VAI has generally been considered cost-effective in most studies [19,57,58]. The clinical benefit and cost-effectiveness of confirming VAI diagnosed on CTA with DSA has not been significantly explored. Given the high false-positive rates in patients with low-grade lesions, DSA may play a role in guiding decisions regarding treatment de-escalation or cessation in these cases. Ultimately, the decision to initiate ACAP empirically in patients with CTA-diagnosed, low-grade VAI vs. pursuing invasive imaging should take into account patient-specific factors, including concurrent polytrauma, the risk of hemorrhage, and the anticipated benefits of therapy.

## 5. Ischemic Complications and Radiographic Outcomes

Posterior circulation stroke is a potentially devastating yet modifiable complication of VAI. Michalopoulos et al. reported a pooled stroke rate of 5% in patients with isolated VAI. However, this figure may underestimate the true risk, as the analysis included series with patients who underwent embolization as part of their management [5]. In contrast, earlier data reported a significantly higher stroke rate of up to 24%, with 8% of deaths attributable to VAI [5,6]. In contemporary clinical practice, the true incidence of ischemic complications likely falls between these two extremes when accounting for the impact of advances in medical therapy and screening protocols.

The Denver grading system serves as a relatively reliable predictor of stroke risk in VAI, with higher-grade lesions (grades III and IV) conferring a higher stroke risk compared to lower-grade lesions (grades I and II) [5,59]. Additionally, there is a substantial but often under-recognized burden of clinically silent ischemia. One study reported that up to 43% of patients with traumatic cerebrovascular injury had new ischemic lesions on delayed routine MRI [60].

Bilateral VAI also represents another important risk factor and is associated with a markedly higher stroke rate compared to isolated lesions (33% vs. 5%) [5]. Furthermore, penetrating cervical trauma has been shown to result in a higher incidence of VAI and a greater proportion of high-grade injuries compared to blunt mechanisms [10]. Although the stroke rate was also higher in the penetrating group (26% vs. 14%), this difference did not reach statistical significance [10].

Concomitant spinal cord injury may be associated with the risk of ischemic stroke [61,62]. In a study by Torina et al., VAI-associated strokes were significantly more frequent in patients with motor-complete spinal cord injury (American Spinal Injury Association Impairment scale [ASIA] A and B) compared to those with a motor-incomplete (ASIA C and D) or neurologically intact (ASIA E) status [62]. Data on the relative risk of stroke secondary to traumatic VAI compared to carotid artery injury are mixed, although a recent meta-analysis reported a higher incidence of stroke in carotid artery injury [59,63,64]. Importantly, the presence of collateral vessels identified on angiography appears to protect against posterior circulation stroke in patients with blunt VAO [65]. Additionally, patients who experienced stroke were more likely to have a higher degree of luminal stenosis than those who did not [66].

The dynamic nature of VAI is increasingly recognized as a potentially important factor that can influence stroke risk [67]. In 2014, Wagenaar et al. reviewed the early repeat angiography (defined as within 10 days) of 255 patients initially presenting with VAI and found that the injury resolved in 56% of grade I, 17% of grade II, 14% of grade III, and 3% of grade IV injuries [68]. A further retrospective analysis of repeat imaging for high-grade VAI at two-month follow-up found that 39% of grade III VAI was radiographically stable, while 56% either resolved or improved [69]. In VAO cases (grade IV), 65% of injuries demonstrated persistent occlusion, while 35% either showed radiographic improvement or resolution. The relatively high rates of spontaneous improvement among low-grade lesions appear consistent across studies [70].

Among VAI patients who initially presented without stroke symptoms, patients with multifocal arterial injuries or occlusive lesions were at a higher risk of developing delayed symptoms after presentation [63]. The greatest risk of developing ischemic symptoms appears to be in the time period immediately surrounding presentation (typically within the first day) [71]. After this window, 98% of patients did not develop a new focal neurological deficit [71]. Although ACAP is the mainstay of medical management, current data are inconclusive regarding its effect on radiographic outcomes in VAI [72].

A recent systematic review and meta-analysis from our group estimated an overall recanalization rate of 38% among VAO patients with subsequent radiographic follow-up [4]. Remarkably, when only studies reporting on cervical spine intervention (fusion or cervical traction) were considered, the rate of spontaneous recanalization post-surgery was as high as 57%. While data on post-recanalization stroke in these studies were insufficient to pool effect sizes, the resolution of intraluminal thrombus in VAI has previously been linked to the development of stroke [66]. Notably, five of nine patients with VAO recanalization from the Indo et al. cohort developed ischemic complications, including one patient who later died from basilar artery occlusion [73]. Other series did not report delayed stroke events associated with VAO recanalization [50,74,75,76].

## 6. Management

### 6.1. Medical Management

Medical management, including antiplatelet, anticoagulation, or combination therapy, is the most common first-line treatment strategy for VAI. A systematic review and meta-analysis of 17 articles totaling 475 patients found that medical management accounted for 56% of all cases, with 16% of patients being managed with antiplatelet alone, 18% of patients with anticoagulation alone, and 26% of patients with a combination therapy [46]. Surgical or endovascular management was utilized in 26% of cases, and expectant management in 18% of cases.

To date, only two randomized controlled trials comparing medical therapies for cervical artery dissection have been conducted, both of which reported no evidence concerning the superiority of either antiplatelet or anticoagulation [77,78]. The Cervical Artery Dissection in Stroke Study (CADISS) trial included a cohort in which 53% of patients had VAI, while the Aspirin versus Anticoagulation in Cervical Artery Dissection (TREAT-CAD) trial included 35% VAI patients. However, these studies did not report the outcomes specific to the VAI subgroup or investigate dissections specific to the trauma context. Several retrospective analyses reported a similar improvement in stroke rates among patients on medical therapy compared to patients with expectant management [79,80,81,82,83]. In the trauma setting, it is often challenging to discern whether the observed improvements are attributable to therapy-associated risk modification or represent the natural history of comorbid injury.

There is some evidence suggesting that treatment delay beyond the first 24 h after presentation may not significantly worsen outcomes [84]. Additionally, some have also advocated for the early resumption of anticoagulation after surgical intervention. In a review of 63 trauma patients requiring anterior or posterior decompression and fusion, Camillo et al. identified 14 patients with concomitant traumatic cerebrovascular injury requiring anticoagulation and reported no perioperative complications related to the anticoagulation use [85]. In this protocol, patients were initiated on low-intensity heparin drip with a goal partial thromboplastin time (PTT) that ranged between 40 and 60 s. Anticoagulation was held two hours before the operation and resumed four hours post-operatively. Due to limited high-quality evidence and concerns regarding hemorrhagic complications, this approach remains controversial and has not been widely adopted in modern clinical practice.

In 2020, the Eastern Association for the Surgery of Trauma (EAST) released an updated practice management guideline for the evaluation and management of BCVI [86]. Based on the available evidence, the panel recommended initiating antithrombotic therapy as early as safely possible to reduce the risk of stroke and mortality. However, the authors did not specify a preferred type of antithrombotic agent, citing a lack of high-quality data regarding the superiority of one agent over another. Table 2 summarizes the individual studies used by EAST to inform their 2020 recommendations. Where possible, data specific to VAI were extracted for this table. Several studies—Biffl et al. (1998), Edwards et al., Fabian et al. and Wahl et al.—were excluded from the table, as they focused exclusively on carotid artery injuries [87,88,89,90]. Similarly, the Eachempeti et al. cohort was also omitted due to the inclusion of only two isolated VAI cases [88].

Although the early trauma cohorts largely favored anticoagulation therapy, more recent studies focusing specifically on VAI continue to show a preference for antiplatelet agents, particularly aspirin [69,72,91]. The duration of ACAP in the VAI population remains unstudied. Given the complexity of polytrauma cases and the lack of high-quality evidence, decisions regarding the choice of agent, the timing of initiation, and the duration of therapy should be tailored to individual patients and their clinical status.

**Table 2:** Studies containing vertebral artery injury used by the Eastern Association for the Surgery of Trauma (EAST) for practice management guidelines regarding the use of antithrombotic therapy.

Study	AC Alone	AP Alone	ACAP	Other	Stroke	Therapy Duration	Note
Biffl 2000 [6]	63% (24/38)	16% (6/38)	0% (0/38)	21% (8/38)	26% (10/38)	Not mentioned	BCVI
Callcut 2012 [92]	30% (22/73)	30% (22/73)	1% (1/73)	38% (28/73)	27% (21/77)	Not mentioned	BCVI + Neurologically Injured Patients
Cothren 2009 [79]	45% (192/422)	21% (90/422)	0% (0/422)	33% (140/422)	11% (45/422)	Not mentioned	BCVI
Miller 2001 [80]	62% (31/50)	26% (13/50)	0% (0/50)	12% (6/50)	14% (7/50)	Not mentioned	VAI only
Stein 2009 [82]	10% (15/147)	36% (53/147)	18% (27/147)	35% (52/147)	12% (18/147)	Not mentioned	BCVI

AC: anticoagulant; AP: antiplatelet; ACAP anticoagulant and/or antiplatelet; BCVI: blunt cerebrovascular injury; VAI: vertebral artery injury.

### 6.2. Endovascular Management

The endovascular management of blunt vertebral artery injury is more commonly described in higher-grade lesions such as pseudoaneurysms or VAO. When feasible, reconstructive strategies are generally preferred in grade III injuries. In a series of 10 patients with pseudoaneurysm, Mei et al. reported favorable outcomes following endovascular treatment with stent-assisted coiling or stenting alone, with no neurological deficits observed [93]. Similarly, Cohen et al. described the successful treatment of nine patients with vertebral pseudoaneurysm using flow diversion, stent-assisted coiling, or stenting alone [94]. The authors further outlined an algorithm for acute vs. delayed intervention and recommended that patients be treated in the acute phase only if angiography showed the significant stenosis of a dominant vertebral artery. Delayed treatment was performed in cases of lesional progression on follow-up imaging, ischemic complications, or patient preference [94]. Pham et al. reviewed six reported cases of endovascular stenting for traumatic VAI, where the indications for intervention included pseudoaneurysm progression to rupture, dissection severity, or contraindication to anticoagulation [95]. Likewise, Lee et al. described five cases of blunt vertebral pseudoaneurysm treated with endovascular techniques—three with stenting and two with stent-assisted coiling—all resulting in the preservation of the parent artery and no neurological complication at follow-up [96].

The improvement of neurological deficit after endovascular therapy has been observed. In a series of 23 vertebral artery dissections, Moon et al. reported that 44% of patients who underwent endovascular management experienced the complete resolution of disability at follow-up [97]. Similar to medical management, determining the extent to which recovery is influenced by the natural history of comorbid injuries remains challenging.

In contrast to grade III injuries, a deconstructive technique is typically the preferred endovascular strategy for VAO, particularly in patients with co-dominant vertebral arteries or with a concurrent need for cervical spine surgery [98]. In three series totaling 27 cases where VAO was managed with endovascular embolization prior to cervical surgery, no postoperative strokes were reported [73,99,100]. Of the retrospective studies examining pre-operative embolization, only Indo et al. included historical cases where pre-operative embolization was not performed. The authors reported a significantly lower stroke rate among the embolized patients compared to the non-embolized patients, both radiographically and clinically (55.6% radiographic stroke and 33.0% symptomatic stroke in the non-embolized group vs. none in the embolized group) [73].

### 6.3. Open Surgical Management

Given the advances in endovascular techniques and their relatively favorable safety profile, open surgical approaches to treating VAI are generally reserved for patients with uncontrolled hemorrhage. Open surgical management begins with stopping the active bleeding by either tamponading the damaged vessel with hemostatic agents, ligation, or clipping. The selection of the hemostatic agent is typically limited to materials with a large surface area such as Gelfoam (Pfizer, New York, NY, USA), Surgicel (Johnson & Johnson, Warsaw, IN, USA), or bone wax, due to concerns regarding the association of distal embolization with liquid hemostatic agents applied directly to the arteriotomy.

The location of the primary lesion dictates how amenable it may be to open surgical repair. The V1 segment is most amenable to open treatment given its pre-osseous course in the neck. In contrast, the V2 and V3 segments are difficult to expose given their course through the transverse foramina. In these regions, brisk bleeding combined with limited visualization can complicate hemostasis efforts and increase the risk of complications such as cervical nerve root injury.

After adequate hemostasis is obtained, follow-up angiographic evaluation is performed to assess the efficacy of treatment [8]. At this point, the decision between open vs. endovascular intervention is made, taking into account the level of injury, hemodynamic stability, and the anatomy of the patient. Further treatment may involve endovascular coiling, surgical clipping, repair, or ligation [101]. The need for repair after VAI remains a topic of debate. VAI isolated to one artery may not warrant a reconstructive repair due to collateral blood flow from the contralateral vertebral artery in a typical co-dominant anatomy [102]. However, a reconstructive approach may be necessary in individuals with hypoplasia of the contralateral vertebral artery [103].

Open vertebral artery reconstructions have become increasingly uncommon in the era of endovascular therapy. In the largest series to date, Berguer et al. [104] described a range of surgical techniques for multiple segments of the vertebral artery. The majority of patients (72%) underwent proximal (V1 segment) reconstructions, most commonly through the transposition of V1 to the common carotid artery (CCA). A smaller subset (12%) required subclavian to V1 bypass grafting due to ipsilateral CCA disease. For distal (V3 segment) reconstruction, the most frequently employed technique involved a saphenous vein bypass from the CCA to V3, with an alternative technique being direct transposition to the external carotid artery (ECA). Similarly, Kieffer et al. reported using saphenous vein grafts for distal vertebral artery bypass in most of their cases [105]. The authors reported a 7% early post-operative occlusion rate, with durable long-term primary patency rates of 89% at 5 years and 88% at 10 years.

**Figure 1:**
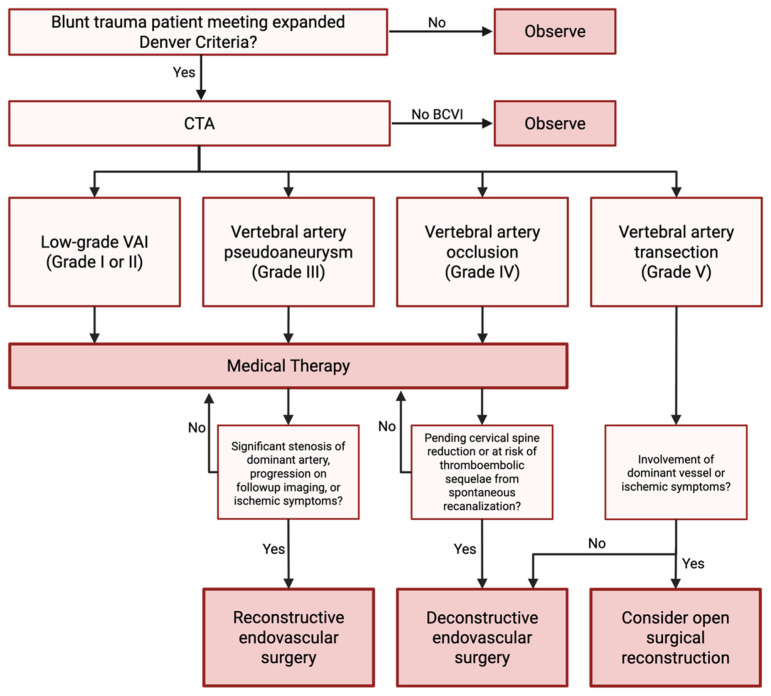
Suggested management algorithm for blunt traumatic vertebral artery injury.

Figure 1 summarizes the suggested management algorithm for blunt traumatic VAI.

## 7. Future Directions

Significant heterogeneity persists in the literature regarding screening, follow-up protocols, and management strategies for VAI. Because the natural history of VAI appears to depend not only on the initial lesion characteristics (e.g., Denver grade) but also subsequent management (i.e., ACAP use and/or cervical intervention), future studies should clearly delineate temporal relationships for individual patients, documenting the sequence and interaction of medical or surgical interventions, with radiographic and clinical follow-up. Additionally, advanced imaging could be used to investigate the rates of infarcts associated with VAI given the subtle and often vague symptomatology of posterior circulation strokes.

## 8. Conclusions

VAI is a known complication of blunt cervical trauma with the potential for significant ischemic events. The widespread adoption of CTA-based screening has not only greatly improved the detection rates but also highlighted the dynamic nature of these lesions over time. Medical management with antiplatelet or anticoagulation therapy remains the mainstay of treatment, particularly for low-grade injuries, and is generally regarded as effective in reducing thromboembolic complications. In recent years, endovascular intervention has demonstrated favorable outcomes in cases with lesional progression, high-grade injuries, failure or contraindication to medical therapy, and more recently, pre-operatively in VAO patients prior to cervical surgery. Given the substantial variability in the management approaches and reporting standards, future studies would benefit from more consistent and detailed documentation, outlining clear temporal relationships between any intervention and radiographic and clinical outcomes.

## Figures and Tables

**Table 1:** Biffl/Denver grading for cerebrovascular injuries [1].

*Grade*	Definition
*I*	Dissection/luminal irregularity, with <25% stenosis
*II*	Dissection/intramural hematoma, with ≥25% stenosis, intraluminal thrombus, or raised intimal flap
*III*	Pseudoaneurysm
*IV*	Occlusion
*V*	Transection

## Data Availability

All data included are available in a publicly accessible repository. Further inquiries can be directed to the corresponding author.

## Abbreviations

The following abbreviations are used in this manuscript:

ACAP	Anticoagulant and/or antiplatelet therapy
ASIA	American Spinal Injury Association
BCVI	Blunt cerebrovascular injury
CTA	Computed tomography angiography
DSA	Digital subtraction angiography
EAST	Eastern Association for the Surgery of Trauma
GCS	Glasgow Coma Scale
MRA	Magnetic resonance angiography
TBI	Traumatic brain injury
VAI	Vertebral artery injury
VAO	Vertebral artery occlusion
